# Vitamin D status among long-term survivors of testicular cancer

**DOI:** 10.18632/oncotarget.14167

**Published:** 2016-12-24

**Authors:** Giuseppe Schepisi, Silvia De Padova, Emanuela Scarpi, Cristian Lolli, Giorgia Gurioli, Cecilia Menna, Salvatore L. Burgio, Lorena Rossi, Valentina Gallà, Valentina Casadio, Samanta Salvi, Vincenza Conteduca, Ugo De Giorgi

**Affiliations:** ^1^ Istituto Scientifico Romagnolo per lo Studio e la Cura dei Tumori (IRST) IRCCS, Via P. Maroncelli, 40 I-47014 Meldola (FC), Italy

**Keywords:** germ cell tumor, testicular cancer survivors, 25-hydroxyvitamin D, testosterone, hypogonadism

## Abstract

A correlation between disturbances in hormone levels and the onset of metabolic disorders has been reported in long-term survivors of testicular cancer (TC).

We evaluated serum vitamin D levels and other biological parameters in a consecutive series of 61 long-term (≥3 years) unilateral TC survivors with a median a follow-up of 4 years and in a cohort of healthy males. Deficient vitamin D levels were observed in 10 (17%) of the 58 long-term unilateral TC survivors but were not reported in healthy males (*p*=.019, Fisher test). Median vitamin D levels were 18.6 ug/L in 58 assessable TC survivors and 23.6 ug/L in 40 healthy males (*p*=.031). In univariate logistic regression analysis, TC diagnosis was associated with inadequate levels of vitamin D (*p*=.047). Vitamin D levels were lower when follow-up was > 10 years, albeit this difference was not statistically significant (*p*=.074). Long-term (especially > 10 years) TC survivors may have difficulty maintaining optimal vitamin D levels. Larger studies are needed to better characterize vitamin D status and possible correlations with premature hormonal aging reported in long-term TC survivors.

## INTRODUCTION

Testicular cancer (TC) is the most common solid tumor in males aged between 15 and 35 years and is a highly curable neoplasm, with nearly 95% of patients cured by orchiectomy and chemotherapy and, when needed, radiotherapy [1, 2]. This excellent outcome is, however, associated with considerable long-term morbidity, including second malignant tumors, cardiovascular disease, neurotoxicity, nephrotoxicity, pulmonary toxicity, hypogonadism, decreased fertility, and psychosocial problems [3, 4]. Little information is available on metabolic disorders in long-term survivors [3].

Vitamin D has various functions in the human organism, including that of bone, calcium and phosphate homeostasis, but also plays a role in male reproduction [5]. Specific vitamin D receptors (VDR) and several enzymes involved in vitamin D metabolism are expressed in germ cells, Sertoli cells, Leydig cells, spermatozoa and epithelium of the human reproductive tract [6], suggesting that male reproduction (not only in humans) strongly correlates with adequate levels of vitamin D. Several effects of this correlation are known, *e.g*. vitamin D increases fertility and regulates the morphology and motility of spermatozoa [8, 9]. Not only VDR expression is needed, but also CYP24R1 expression is considered to be indicative of good quality sperm; CYP24R1, expressed in mouse and human testis, is considered the putative enzyme for vitamin D hydroxylation [10]. CYP24R1positive spermatozoa are also positively correlated with sperm count, morphology, concentration and motility [6]. Vitamin D levels are thought to be correlated with malignant transformation into TC because 1-25 di-hydroxyvitamin D_3_ promotes differentiation and decreases proliferation of different tumor types (not only TC, but also breast and kidney cancer) [7, 8]. Moreover, a specific correlation has been found between VDR, enzymes involved in vitamin D metabolism and cellular differentiation: high levels are observed in carcinoma in situ, whereas low levels are found in invasive seminoma. [6]. In a study by Foresta et al., male patients submitted to radical orchiectomy for bilateral TC showed significantly lower concentrations of 25-hydroxyvitamin D than healthy controls. The authors concluded that testiculopathy, no matter what the cause, may be involved in the pathogenesis of vitamin D insufficiency through impaired 25-hydroxylase activity [12]. An association between unilateral TC and alteration of bone status, even in the presence of unvaried androgen and estrogen levels, was also recently hypothesized [13].

A correlation between disturbances in hormone levels and the development of metabolic disorders has been reported in long-term (*i.e*. follow up > 3 years) TC survivors [14, 15].

The aim of the present study was to evaluate vitamin D levels in a cohort of Italian long-term TC survivors and to investigate potential associations with patient characteristics and other metabolic parameters.

## RESULTS

Three of the 61 patients had undergone bilateral orchiectomy and 58 unilateral orchiectomy. In the group with bilateral tumors, one patient showed deficient vitamin D levels (6.8 μg/mL) and the remaining 2 insufficient/inadequate levels (20 μg/mL and 22.5 μg/L, respectively). Among the 58 participants with unilateral TC, 11 (19.0%) had normal serum vitamin D values, while 47 (81.0%) had suboptimal levels, 10 (17.2%) of whom with deficient levels, 23 (39.7%) with insufficient levels and 14 (24.1%) with inadequate levels. Deficient vitamin D levels were observed in 10 (17%) of the 58 long-term unilateral TC survivors but were not reported in healthy males (*p*=.019, Fisher test). A significant difference was found in median vitamin D levels between long-term TC survivors and healthy males, *i.e*. 18.6 μg/mL (range 6.9- 58.2) vs. 23.6 (range 10.0-49.1), respectively (*p*=.031). The two groups were similar in terms of median age [37 years (range 21-68) vs. 38 years (range 25-51), respectively) and season of measurement.

Median age of the unilateral TC group was 37 years (range 21- 68) and median follow-up was 4 years (range 3-10). Vitamin D supplementation was given to those patients with deficient levels by primary care physicians or by oncologists. Vitamin D supplementation was given also to three patients with inadequate vitamin D levels by primary care physicians.

The clinical characteristics of the 58 long-term unilateral TC survivors and previous treatments are summarized in Table 1. Among the 40 healthy males, 38 of them had sufficient Vitamin D levels, the remaining 2 males had inadequate/borderline levels, no case with deficient levels were reported. The nonparametric Spearman's correlation test revealed an inverse correlation between vitamin D levels and PTH (*p*=0.002, significant after Bonferroni correction for multiple testing). Furthermore, PTH concentrations correlated with phosphoremia (*p*=.041) and testosterone (*p*=.041), while testosterone correlated with estradiol levels (*p*=.002, significant after Bonferroni correction for multiple testing) (Table 2). FSH and LH were not correlated with vitamin D levels. Means values of the parameters evaluated in patients are described in Table 3. We also evaluated the correlation between metabolic syndrome parameters, such as hypertension and dyslipidemia, and vitamin D levels in unilateral TC group. Hypertension was reported in only one (2%) patient with insufficient vitamin D, whereas dyslipidemia was found in 6 (10%) patients, 4 of whom had insufficient and 2 inadequate vitamin D levels. None of these diseases was found in the group of healthy males. No correlation was observed between these parameters and QoL (data not shown). No correlation was found between vitamin D levels and previous treatment with orchiectomy only (n=17, 29%) and orchiectomy plus radiotherapy and/or chemotherapy (n=41, 71%).

**Table 1:** Patient characteristics (n=58)

Characteristics	N° (%)
Median age, years (range)	37 (21 to 68)
Histology	
Seminoma	29 (50%)
Nonseminoma	29 (50%)
Stage at diagnosis	
Stage I	40 (69%)
Stage II	11 (19%)
Stage III	7 (12%)
Previous therapy	
Surgery only	17 (29%)
Orchiectomy and chemotherapy	39 (67%)
Orchiectomy and radiotherapy	2 (3%)
Risk Factors	
Hypertension	1 (2%)
Dyslipidemia	6 (10%)
Body mass index >30	1 (2%)

**Table 2:** Vitamin D levels in unilateral testicular cancer survivors

	Calcemia		Phosphoremia		PTH		Testosterone		Estradiol		Calcitonin	
	r_s_	*p*	r_s_	*p*	r_s_	*p*	r_s_	*p*	r_s_	*p*	r_s_	*p*
**Vitamin D**	0.24	0.095	0.04	0.776	−0.40	0.003	−0.10	0.450	0.01	0.996	0.05	0.756
**Calcemia**	-	-	0.03	0.829	−0.12	0.427	0.19	0.191	0.16	0.275	0.01	0.978
**Phosphoremia**	-	-	-	-	−0.30	0.041	0.07	0.638	0.19	0.206	0.17	0.273
**PTH**	-	-	-	-	-	-	−0.28	0.041	−0.21	0.147	0.06	0.698
**Testosterone**	-	-	-	-	-	-	-	-	0.42	0.002	−0.15	0.295
**Estradiol**	-	-	-	-	-	-	-	-	-	-	−0.02	0.876

Abreviations: PTH, parathormone; r_s_ = Spearman Rank-order coefficient.

**Table 3:** Means values (standard deviation=SD) of patients

	Mean value (SD)
Vitamin D levels (20-50 mcg/l)	19.84 (10.06)
Calcium levels (8.5-10.2 mg/dl)	9.59 (0.36)
Phosphate levels (2.5 – 4.5 mg/dl)	3.33 (0.59)
PTH (10-55 pg/mL)	41.36 (12.50)
Calcitonin (<16 pg/ml)	3.22 (2.03)
FSH (1.5-12.4 mIU/ml)	15.99 (14.42)
LH (1.8-12.0 mUI/ml)	9.18 (6.69)
Testosterone (4.60 - 31.0 nmol/L)	15.28 (6.54)
Beta-estradiol (10 - 40 pg/ml)	73.35 (32.16)
Progesterone (0.20 - 1.40 ng/ml)	1.57 (0.81)

Abreviations: PTH, parathormone; FSH, follicle-stimulating hormone; LH, luteinizing hormone.

In addition, 8 patients with a follow-up > 10 years (median 13 years, range 10- 24) had a median vitamin D level of 13.1 μg/L (range 7.8-24.6), whereas patients with a shorter follow-up (median 4 years, range 3-10) had a median vitamin D level of 19.5 μg/mL (range 6.8-58.3). The difference was not statistically significant (*p*=.074). In univariate logistic regression analysis, a diagnosis of TC was associated with inadequate levels of vitamin D (odds ratio= 1.04, 95% CI 1.01-1.09) (*p*=.047).

## DISCUSSION

Vitamin D is important for humans because of its role in regulating blood pressure and cell growth and in preventing cancer progression [16–18]. Insufficient vitamin D levels are correlated with diabetes, autoimmune diseases, congestive heart failure and multiple sclerosis [19, 20]. Serum vitamin D levels in the body depend on two factors, the most important being sun exposure, especially ultraviolet light between 290 and 305 nm. The sun converts 7-dehydrocholesterol into the biologically inactive cholecalcipherol D3 which becomes active via 2 hydroxylation steps, first in the liver and then in the kidneys [6]. However, the term vitamin D does not only refer to cholecalcipherol but also to ergocalcipherol (D2), which is produced from plant sterols. Thus, another important source of vitamin D is diet and this source is particularly important in countries with insubstantial sunshine. In fact, at latitudes >40°, sunlight is not strong enough to obtain conversion to D3 during winter [21]. A study conducted on indigenous people of northern Russia reported that their traditional diet guaranteed sufficient vitamin D levels. Conversely, the same people who abandoned their nomadic culture to follow a western lifestyle (including diet) showed lower serum levels of vitamin D [22]. Vitamin D is also an essential element in male fertility, several studies highlighting its role in regulating the morphology and motility of spermatozoa (9,10). There is worldwide consensus that blood 25(OH)D levels below 25 nmol/l (or 10 μg/mL) can be considered ‘deficient’ [23].

In our study, we found a high percentage of deficient levels of vitamin D in TC patients group, in agreement with results from other studies conducted on similar populations [24]. This high percentage seems to be associated with follow up longer than 10 years, suggesting that other larger studies in this cohort of TC survivors are needed. It is worthy of note that, although a diagnosis of TC was associated with inadequate levels of vitamin D (odds ratio 1.04, *p*=.047) and 17% of unilateral TC long-term survivors also showed insufficient vitamin D levels (*p*=.019), no cases of deficient vitamin D levels were observed among the group of healthy males although two borderline cases were found (30 nmol/l). Our control group was composed by males with the same age and without tumor pathology, therefore we cannot exclude possible selection bias. In another study conducted in France in the framework of the French SUVIMAX project, some cases of hypovitaminosis ranging between 0 and 7% were found in Mediterranean coast population; however, this population were not selected for tumor diagnosis [25]. In 2011, Robien et al. [26] published data on vitamin D status in long-term survivors of hematopoietic cell transplantation, observing inadequate vitamin D levels in 35% of patients, even though the majority of patients reported a regular use of vitamin D supplements. This finding is also valid for unilateral TC survivors because high-dose chemotherapy with hematopoietic cell transplantation is also a salvage treatment option for these patients [27–29]. More recently, Nappi *et al*. [30] reported that, in a group of 78 TC patients of whom 83% had insufficient (< 30μg/mL) and 43% had deficient (< 20 μg/ml) 25-OH vitamin D levels, these data were not influenced by time from diagnosis or treatment received. In our results, no impact of chemotherapy and systemic treatments was also shown. In addition, Willemse *et al*. [31] reported that metastatic TC survivors had significant bone loss during the first year after chemotherapy and that this condition was maintained even after 5 years of follow-up.

Another important finding of our study was that vitamin D serum levels were lower in TC patients with abnormal testosterone and/or LH serum levels. Specifically, we noticed a trend in favor of a decrease in vitamin D levels in patients with a long-term follow-up (> 10 years) (median 13 years, range 10-24) compared to those with a shorter follow-up (*p*=.074). Sprauten et al. observed that patients with a >10-year follow-up were also at risk of hypogonadism [32]. These authors found that long-term TC survivors were at risk of premature hormonal aging and were also more vulnerable to age-related deterioration of sex hormones than controls, especially after intensive TC treatment. Another research by Ondrusova et al. in a cohort of 879 TC survivors showed that in 823 patients with unilateral TC, a testosterone deficiency was found in 19.5% of cases, with an increased LH in 19.1%, and bone damage (osteopenia and/or osteoporosis) in 50.6% of cases [33]. Taking into account these considerations, our observation of lower vitamin D levels in long-term unilateral TC survivors would seem to be related to the initial hypogonadism observed in these patients.

Our study has a number of limitations, one of which was the small number of patients involved and the poor number of controls. Data were collected after a disease-free status of ≥ 3 years as vitamin D deficits tend to appear after this period, but not before treatment because of the retrospective nature of our evaluation. Moreover, as data were only collected during winter months, we were not able to study seasonal variations in vitamin D levels. Several studies have, on the other hand, reported that a vitamin D deficit is closely correlated with seasonal variations in serum testosterone concentrations [34, 35].

Patients submitted to orchiectomy may have difficulty in maintaining optimal vitamin D blood levels, especially those with >10-year survival. Larger studies are needed to better characterize vitamin D status and possible correlations with premature hormonal aging reported in long-term TC survivors.

## MATERIALS AND METHODS

### Patient population

Between November 2013 and April 2015, 61 patients who had been treated at our institute for TC and who were still disease-free ≥ 3 years after the end of treatment were enrolled onto the study. The follow-up time was considered by the time when patients were disease-free, that is by orchiectomy in patients treated with orchiectomy alone and/or followed by adjuvant therapies or surveillance, and by the end of treatment for metastatic patients. Fifty-eight patients had undergone unilateral orchiectomy and 3 bilateral orchiectomy.

Serum levels of vitamin D, PTH, calcium, phosphorus, calcitonin, testosterone, LH, FSH and beta-estradiol, as well as metabolic syndrome parameters such as cholesterol, glycemia and hypertension were analyzed from November to March in all participants. No pre-treatment determination of vitamin D levels was performed. Data on diet, lifestyle and medical history were collected. Patients were also asked to complete the Medical Outcomes Study Short Form 36 (SF-36) to assess their health-related quality of life (HRQoL). The SF-36, known for its comprehensiveness, brevity, and high standards of reliability and validity, measures two major health concepts (physical and mental health) with 36 items, generating eight multi-item scales: physical functioning, role limitations due to physical problems, bodily pain, general health perceptions, vitality, social functioning, role-limitations because of emotional problems, and mental health [36, 37]. In each subscale, a higher score indicates a more favorable QoL [38]. We used the Italian version of the SF-36 questionnaire [37]. We also recruited a cohort of 40 healthy males as controls, matched to the 61 disease-free survivors. Healthy individuals were recruited from hospital laboratory and clinical staff, and none had been previously clinically diagnosed with any type of cancer. Serum levels of vitamin D were analyzed in the cohort of healthy males.

In accordance with international guidelines [23], we classified suboptimal serum levels of vitamin D as follows: sufficient (>50 to ≤75 nmol/L = >20 to ≤30 μg/mL); inadequate (>25 to ≤50 nmol/L = >10 to ≤20 μg/mL); deficient (≤25 nmol/L = ≤10 μg/mL). No participant has ever carried out vitamin D supplementation prior to study entry. The study was approved by IRST Ethics Committee and written informed consent was obtained from all patients.

### Statistical analysis

The relationship between vitamin D levels and clinical pathological or biological variables was analyzed using a non-parametric ranking statistic (Wilcoxon-Mann-Whitney median test). Spearman's correlation test was used to investigate the relationship between vitamin D levels and other parameters considered as continuous variables. The Chi-square or Fisher test was used to evaluate the association between vitamin D status and serum levels of LH and testosterone. Two-sided P < 0.05 before Bonferroni correction were considered as nominally significant. After applying a Bonferroni's correction for multiple testing, p-values <0.0025 was considered as significant.

All statistical analyses were carried out with SAS Statistical Software (version 9.4, SAS Institute, Cary, NC, USA).
